# Supplementary material for: Comprehensive Targeted Metabolomic Study in the Lung, Plasma, and Urine of PPE/LPS-Induced COPD Mice Model
Source: Int J Mol Sci. 2022 Mar 2;23(5):2748. doi: 10.3390/ijms23052748 (PMC8911395; doi:10.3390/ijms23052748)
Supplement: Supplementary file 1 [file ijms-23-02748-s001.zip › ijms-1596269-supplementary.pdf]

Supplementary Material

Comprehensive targeted metabolomic study in the lung, plasma, and urine of PPE/LPS-induced COPD mice model

Hyeon-Young Kim [1,2,†], Hyeon-Seong Lee [3,4,†], In-Hyeon Kim [1,2], Youngbae Kim [5], Moongi Ji [5], Songjin Oh [5], Doo-Young Kim [5,6], Wonjae Lee [3], Sung-Hwan Kim [1,#] and Man-Jeong Paik [5,*]

[1] Jeonbuk Branch Institute, Korea Institute of Toxicology, Jeongeup 56212, Republic of Korea; hyeonyoung.kim@kitox.re.kr (H-Y.K); inhyeon.kim@kitox.re.kr (I-H.K)
[2] College of Veterinary Medicine, Chonnam National University, Gwangju 61186, Republic of Korea
[3] College of Pharmacy, Chosun University, Gwangju, Republic of Korea; dlrudgks1022@kist.re.kr (H-S.L); wlee@chosun.ac.kr (W.L)
[4] Korea Institute of Science and Technology, Gangneung Institute of Natural Products, Gangneung-si, Republic of Korea
[5] College of Pharmacy, Sunchon National University, Suncheon, Republic of Korea; unkr2003@naver.com (Y.K); wlansrl@naver.com (M.J); osj7797@naver.com (S.O), 20110074@hdpharm.co.kr (D-Y.K)
[6] Hyundai Pharm, New Drug Discovery Lab, Yongin, Republic of Korea

† These authors contributed equally to this work.
* Correspondence: paik815@sunchon.ac.kr; 82-61-750-3762
\# Correspondence: sunghwan.kim@kitox.re.kr; 82-63-570-8757

Supplementary Table S1. Statistical results of determined metabolites in the lung between COPD1 and VC1 or COPD2 and VC2.

Name	log_2FC(COPD1/VC1)	p-value of COPD1 and VC1	FDR of COPD1 and VC1	VIP in PLS-DA of COPD1 and VC1	log_2FC(COPD2/VC2)	p-value of COPD2 and VC2	FDR of COPD2 and VC2	VIP in PLS-DA of COPD2 and VC2
1-Methylhistidine	1.37	2.17E-05	6.22E-05	1.32	0.79	1.30E-04	3.45E-04	1.27
2-Hydroxyglutaric acid	0.07	3.53E-01	4.06E-01	0.02	-0.25	1.23E-01	1.66E-01	0.55
3-Hydroxybutyric acid	-0.07	7.39E-01	7.61E-01	0.02	0.64	3.53E-01	4.27E-01	0.41
3-Methylhistidine	1.87	1.08E-05	4.15E-05	1.21	1.09	1.08E-05	4.67E-05	1.29
4-Hydroxy-L-proline	1.03	1.08E-05	4.15E-05	1.36	0.90	1.08E-05	4.67E-05	1.43
4-Hydroxyphenylacetic acid	-0.57	1.08E-05	4.15E-05	1.38	-0.53	2.17E-05	7.11E-05	1.35
4-Hydroxyphenyllactic acid	-0.56	1.08E-05	4.15E-05	1.40	-0.53	1.08E-05	4.67E-05	1.37
a-Aminoadipic acid	1.05	1.08E-05	4.15E-05	1.42	0.65	1.08E-05	4.67E-05	1.41
a-Ketoglutaric acid	-0.15	2.32E-02	3.48E-02	0.78	-0.43	3.89E-03	7.66E-03	0.85
Alanine	0.92	1.08E-05	4.15E-05	1.28	0.38	4.33E-05	1.36E-04	1.29
a-Linolenic acid	0.12	7.96E-01	8.08E-01	0.33	0.05	7.39E-01	7.97E-01	0.23
Arachidonic acid	0.44	1.05E-03	2.13E-03	1.07	0.58	2.88E-03	5.84E-03	0.90
Arginine	-0.19	2.32E-02	3.48E-02	0.72	-0.04	2.18E-01	2.83E-01	0.26
Asparagine	1.30	1.08E-05	4.15E-05	1.21	0.58	1.08E-05	4.67E-05	1.36
Aspartic acid	1.20	1.08E-05	4.15E-05	1.36	0.64	2.17E-05	7.11E-05	1.44
b-Alanine	-0.16	1.47E-02	2.30E-02	0.95	-0.24	1.50E-03	3.24E-03	1.12
cis-Aconitic acid	-0.12	6.30E-02	8.36E-02	0.53	-0.42	1.47E-02	2.53E-02	0.83
Citric acid	-0.08	2.18E-01	2.59E-01	0.29	-0.85	1.50E-03	3.24E-03	1.07
Citrulline	0.41	5.24E-02	7.09E-02	0.79	0.03	5.29E-01	6.18E-01	0.24
Creatine	0.50	3.25E-04	7.23E-04	1.15	0.38	2.17E-05	7.11E-05	1.34
Cysteine	0.86	7.58E-05	2.01E-04	1.25	0.36	4.87E-04	1.20E-03	1.21
Cystine	-0.23	8.92E-02	1.10E-01	0.64	-0.23	8.92E-02	1.23E-01	0.74
Docosahexaenoic acid	0.07	6.31E-01	6.59E-01	0.23	0.00	7.96E-01	8.45E-01	0.13
Docosapentaenoic acid	0.41	3.89E-03	6.70E-03	0.96	0.26	3.55E-02	5.56E-02	0.77
Eicosanoic acid	-0.67	4.33E-05	1.20E-04	1.27	-0.48	7.58E-05	2.27E-04	1.20
Eicosapentaenoic acid	0.62	2.09E-03	3.90E-03	0.95	0.38	3.55E-02	5.56E-02	0.76
Eicosenoic acid	-0.33	2.09E-03	3.90E-03	0.95	-0.16	1.65E-01	2.20E-01	0.55
Fumaric acid	0.15	7.53E-02	9.80E-02	0.68	-0.15	1.00E+00	1.00E+00	0.36
g-Aminobutyric acid	1.05	2.17E-05	6.22E-05	1.29	1.26	1.08E-05	4.67E-05	1.45
g-Linolenic acid	0.19	4.33E-02	5.97E-02	0.69	-0.08	2.80E-01	3.51E-01	0.23
Glutamic acid	0.92	1.08E-05	4.15E-05	1.31	0.55	2.17E-05	7.11E-05	1.39
Glutamine	1.31	1.08E-05	4.15E-05	1.29	0.66	1.08E-05	4.67E-05	1.38
GSH	0.33	3.89E-03	6.70E-03	0.96	0.14	7.53E-02	1.06E-01	0.71
Glycine	0.66	1.08E-05	4.15E-05	1.28	0.34	3.25E-04	8.30E-04	1.19
Glycolic acid	0.12	3.93E-01	4.30E-01	0.41	0.05	1.00E+00	1.00E+00	0.13
GSSG	0.72	2.17E-05	6.22E-05	1.19	0.23	7.53E-02	1.06E-01	0.74
Histidine	1.37	2.17E-05	6.22E-05	1.22	0.66	1.08E-05	4.67E-05	1.33
Homocysteine	-0.08	3.53E-01	4.06E-01	0.42	-0.20	2.09E-03	4.37E-03	0.96
Isocitric acid	0.24	1.47E-02	2.30E-02	0.20	0.71	1.50E-03	3.24E-03	0.48
Isoleucine	0.64	1.05E-03	2.13E-03	1.16	0.22	5.20E-03	9.43E-03	0.94
Lactic acid	0.03	9.71E-01	9.71E-01	0.05	-0.06	6.31E-01	7.13E-01	0.17
Leucine	1.46	1.08E-05	4.15E-05	1.38	0.82	1.08E-05	4.67E-05	1.49
Linoleic acid	-0.05	2.18E-01	2.59E-01	0.25	-0.02	7.39E-01	7.97E-01	0.01
L-Targinine	0.51	5.20E-03	8.74E-03	0.95	0.47	1.30E-04	3.45E-04	1.22
Lysine	1.15	2.17E-05	6.22E-05	1.28	0.66	1.08E-05	4.67E-05	1.41
Malic acid	0.21	3.55E-02	4.99E-02	0.46	-0.40	1.05E-03	2.50E-03	1.03
Malonic acid	-0.38	3.89E-03	6.70E-03	0.89	-0.30	2.88E-02	4.73E-02	0.81
Methionine	1.08	1.08E-05	4.15E-05	1.32	0.60	1.08E-05	4.67E-05	1.44
Myristoleic acid	-0.55	1.08E-05	4.15E-05	1.37	-0.59	2.17E-05	7.11E-05	1.38
Nervonic acid	-0.16	3.55E-02	4.99E-02	0.71	-0.08	3.15E-01	3.88E-01	0.28
Octadecanoic acid	-0.35	8.93E-03	1.47E-02	0.88	-0.00	9.12E-01	9.53E-01	0.15
Oleic acid	0.06	4.81E-01	5.11E-01	0.23	-0.01	9.71E-01	9.99E-01	0.04
Ornithine	0.97	1.30E-04	3.32E-04	1.06	0.23	1.85E-02	3.12E-02	0.84
Oxaloacetic acid	0.26	3.55E-02	4.99E-02	0.76	0.03	5.79E-01	6.66E-01	0.06
Palmitic acid	0.18	8.92E-02	1.10E-01	0.62	0.28	8.93E-03	1.58E-02	0.79
Palmitoleic acid	0.45	1.50E-03	2.97E-03	1.03	0.09	2.47E-01	3.16E-01	0.36
Phenylalanine	1.59	1.08E-05	4.15E-05	1.38	1.05	1.08E-05	4.67E-05	1.50
Pipecolic acid	0.16	3.93E-01	4.30E-01	0.34	-0.06	7.39E-01	7.97E-01	0.07
Proline	0.70	2.06E-04	4.73E-04	1.15	0.31	1.30E-04	3.45E-04	1.21
Pyroglutamic acid	-0.08	3.93E-01	4.30E-01	0.37	-0.09	3.93E-01	4.68E-01	0.41
Pyruvic acid	0.41	4.81E-01	5.11E-01	0.21	0.63	4.33E-02	6.49E-02	0.66
Serine	1.35	1.08E-05	4.15E-05	1.31	0.70	1.08E-05	4.67E-05	1.44
Succinic acid	0.23	8.92E-02	1.10E-01	0.63	0.40	4.33E-02	6.49E-02	0.75
Tetracosanoic acid	-0.72	4.87E-04	1.05E-03	1.09	-0.50	5.20E-03	9.43E-03	0.88
Tetradecanoic acid	0.36	2.06E-04	4.73E-04	1.17	0.15	6.30E-02	9.25E-02	0.58
Threonine	0.95	2.17E-05	6.22E-05	1.23	0.49	1.08E-05	4.67E-05	1.36
Tryptophan	1.52	1.08E-05	4.15E-05	1.27	0.82	1.08E-05	4.67E-05	1.39
Tyrosine	1.40	1.08E-05	4.15E-05	1.31	0.81	1.08E-05	4.67E-05	1.45
Valine	0.59	2.06E-04	4.73E-04	1.18	0.25	5.20E-03	9.43E-03	1.02

**Supplementary Table S2.** Result from quantitative metabolite set enrichment analysis in COPD1						
	Total Cmpd	Hits	Statistic Q	Expected Q	Raw p	FDR
Tyrosine metabolism	42	2	78.91	5.26	5.78E-09	2.25E-07
beta-Alanine metabolism	21	3	61.20	5.26	1.66E-08	3.24E-07
Pyrimidine metabolism	39	2	55.86	5.26	5.06E-08	3.98E-07
Lysine degradation	25	2	79.17	5.26	5.09E-08	3.98E-07
Pantothenate and CoA biosynthesis	19	4	61.81	5.26	5.80E-08	3.98E-07
Butanoate metabolism	15	3	57.85	5.26	6.80E-08	3.98E-07
Arginine biosynthesis	14	6	54.17	5.26	7.13E-08	3.98E-07
Nicotinate and nicotinamide metabolism	15	1	80.28	5.26	9.23E-08	4.50E-07
Phenylalanine metabolism	10	2	78.49	5.26	1.79E-07	6.89E-07
Phenylalanine, tyrosine and tryptophan biosynthesis	4	2	78.49	5.26	1.79E-07	6.89E-07
D-Glutamine and D-glutamate metabolism	6	5	64.23	5.26	1.94E-07	6.89E-07
Alanine, aspartate and glutamate metabolism	28	8	60.75	5.26	5.50E-07	1.79E-06
Sphingolipid metabolism	21	1	74.76	5.26	8.77E-07	2.45E-06
Porphyrin and chlorophyll metabolism	30	2	72.93	5.26	8.80E-07	2.45E-06
Ubiquinone and other terpenoid-quinone biosynthesis	9	1	74.53	5.26	9.54E-07	2.48E-06
Nitrogen metabolism	6	2	73.68	5.26	1.11E-06	2.70E-06
Cysteine and methionine metabolism	33	3	72.86	5.26	1.31E-06	3.00E-06
Aminoacyl-tRNA biosynthesis	48	20	68.09	5.26	1.42E-06	3.08E-06
Histidine metabolism	16	4	70.64	5.26	1.68E-06	3.45E-06
Purine metabolism	65	1	72.58	5.26	1.87E-06	3.66E-06
Glyoxylate and dicarboxylate metabolism	32	6	53.30	5.26	2.44E-06	4.53E-06
Primary bile acid biosynthesis	46	1	71.08	5.26	3.05E-06	5.19E-06
Biotin metabolism	10	1	71.07	5.26	3.06E-06	5.19E-06
Selenocompound metabolism	20	1	70.87	5.26	3.26E-06	5.30E-06
Glycine, serine and threonine metabolism	33	5	67.42	5.26	4.00E-06	6.24E-06
Tryptophan metabolism	41	1	70.05	5.26	4.21E-06	6.31E-06
Arginine and proline metabolism	38	6	55.60	5.26	4.85E-06	7.00E-06
Fatty acid biosynthesis	47	2	47.12	5.26	6.28E-06	8.75E-06
Valine, leucine and isoleucine degradation	40	3	67.00	5.26	6.93E-06	9.32E-06
Valine, leucine and isoleucine biosynthesis	8	4	66.77	5.26	7.85E-06	1.02E-05
Biosynthesis of unsaturated fatty acids	36	4	48.28	5.26	8.08E-06	1.02E-05
Taurine and hypotaurine metabolism	8	1	67.58	5.26	8.73E-06	1.03E-05
Thiamine metabolism	7	1	67.58	5.26	8.73E-06	1.03E-05
Glutathione metabolism	28	6	60.49	5.26	2.04E-05	2.34E-05
Arachidonic acid metabolism	36	1	49.89	5.26	5.00E-04	5.57E-04
Propanoate metabolism	23	1	39.13	5.26	3.18E-03	3.44E-03
Citrate cycle (TCA cycle)	20	3	17.68	5.26	1.16E-02	1.22E-02
Glycolysis / Gluconeogenesis	26	1	24.90	5.26	2.51E-02	2.51E-02
Pyruvate metabolism	22	1	24.90	5.26	2.51E-02	2.51E-02

**Supplementary Table S3.** Result from quantitative metabolite set enrichment analysis in COPD2						
	Total Cmpd	Hits	Statistic Q	Expected Q	Raw p	FDR
Pyrimidine metabolism	39	2	62.52	5.26	5.78E-10	1.91E-08
Tyrosine metabolism	42	2	77.29	5.26	9.78E-10	1.91E-08
Butanoate metabolism	15	3	62.46	5.26	2.12E-09	2.29E-08
Phenylalanine metabolism	10	2	86.12	5.26	2.94E-09	2.29E-08
Phenylalanine, tyrosine and tryptophan biosynthesis	4	2	86.12	5.26	2.94E-09	2.29E-08
beta-Alanine metabolism	21	3	67.12	5.26	5.00E-09	3.25E-08
Cysteine and methionine metabolism	33	4	64.08	5.26	6.57E-09	3.66E-08
Histidine metabolism	16	4	73.30	5.26	1.15E-08	5.59E-08
Alanine, aspartate and glutamate metabolism	28	8	66.02	5.26	1.67E-08	7.26E-08
Ubiquinone and other terpenoid-quinone biosynthesis	9	1	83.11	5.26	2.26E-08	8.80E-08
Lysine degradation	25	2	78.29	5.26	4.15E-08	1.47E-07
Glyoxylate and dicarboxylate metabolism	32	7	52.88	5.26	4.73E-08	1.54E-07
Nicotinate and nicotinamide metabolism	15	1	81.28	5.26	5.74E-08	1.60E-07
Sphingolipid metabolism	21	1	81.27	5.26	5.77E-08	1.60E-07
Pantothenate and CoA biosynthesis	19	4	57.43	5.26	6.16E-08	1.60E-07
Biotin metabolism	10	1	78.66	5.26	1.89E-07	4.61E-07
D-Glutamine and D-glutamate metabolism	6	5	66.12	5.26	2.74E-07	6.07E-07
Nitrogen metabolism	6	2	75.59	5.26	2.80E-07	6.07E-07
Tryptophan metabolism	41	1	76.09	5.26	5.34E-07	1.10E-06
Aminoacyl-tRNA biosynthesis	48	19	70.48	5.26	6.22E-07	1.21E-06
Arginine and proline metabolism	38	5	63.32	5.26	7.09E-07	1.31E-06
Arginine biosynthesis	14	5	57.75	5.26	7.63E-07	1.31E-06
Purine metabolism	65	1	75.12	5.26	7.70E-07	1.31E-06
Glycine, serine and threonine metabolism	33	5	67.77	5.26	1.48E-06	2.40E-06
Porphyrin and chlorophyll metabolism	30	2	66.10	5.26	4.93E-06	7.69E-06
Selenocompound metabolism	20	1	66.14	5.26	1.30E-05	1.96E-05
Valine, leucine and isoleucine biosynthesis	8	4	59.04	5.26	2.83E-05	4.09E-05
Glutathione metabolism	28	4	54.40	5.26	3.19E-05	4.45E-05
Biosynthesis of unsaturated fatty acids	36	3	37.78	5.26	7.11E-05	9.56E-05
Valine, leucine and isoleucine degradation	40	3	54.46	5.26	8.19E-05	1.07E-04
Taurine and hypotaurine metabolism	8	1	57.34	5.26	1.11E-04	1.35E-04
Thiamine metabolism	7	1	57.34	5.26	1.11E-04	1.35E-04
Primary bile acid biosynthesis	46	1	56.14	5.26	1.44E-04	1.70E-04
Propanoate metabolism	23	1	49.92	5.26	4.97E-04	5.70E-04
Citrate cycle (TCA cycle)	20	4	27.45	5.26	1.21E-03	1.34E-03
Fatty acid biosynthesis	47	2	25.20	5.26	3.70E-03	4.00E-03
Arachidonic acid metabolism	36	1	32.25	5.26	9.00E-03	9.49E-03
Fatty acid elongation	39	1	24.64	5.26	2.60E-02	2.60E-02
Fatty acid degradation	39	1	24.64	5.26	0.026007	2.60E-02

Supplementary Table S4. Statistical results of determined metabolites in the plasma between COPD1 and VC1 or COPD2 and VC2.

Name	log2FC(COPD1/ VC1)	p-value of COPD1 and VC1	FDR of COPD1 and VC1	VIP in PLS-DA of COPD1 and VC1	log2FC(COPD2/ VC2)	p-value of COPD2 and VC2	FDR of COPD2 and VC2	VIP in PLS-DA of COPD2 and VC2
1-Methylhistidine	0.47	4.33E-02	3.41E-01	1.99	0.185	1.22E-01	4.15E-01	0.47
3-Methylhistidine	0.15	2.80E-01	6.12E-01	0.79	0.152	1.73E-01	4.90E-01	0.15
4-Hydroxy-L-proline	0.06	3.93E-01	6.22E-01	0.19	0.080	5.73E-01	9.68E-01	0.06
5-Hydroxylysine	0.12	9.12E-01	9.84E-01	0.47	-0.396	2.05E-02	2.79E-01	0.12
Alanine	-0.30	1.90E-01	4.98E-01	1.05	-0.020	9.65E-01	9.80E-01	-0.30
a-Aminoadipic acid	-0.24	1.85E-02	2.52E-01	1.62	-0.24	8.31E-02	3.77E-01	-0.24
a-Aminobutyric acid	-0.07	5.29E-01	6.54E-01	0.43	-0.05	8.97E-01	9.68E-01	-0.07
a-Linolenic acid	0.11	4.81E-01	6.54E-01	0.65	0.23	8.31E-02	3.77E-01	0.11
Arachidonic acid	0.22	3.15E-01	6.12E-01	0.31	-0.31	5.15E-01	9.21E-01	0.22
Arginine	0.22	3.53E-01	6.22E-01	0.80	-0.16	3.60E-01	7.65E-01	0.22
Asparagine	0.17	3.93E-01	6.22E-01	0.92	-0.18	1.73E-01	4.90E-01	0.17
Aspartic acid	0.18	1.65E-01	4.89E-01	1.19	0.17	1.01E-01	4.04E-01	0.18
Citrulline	0.06	5.79E-01	6.79E-01	0.53	-0.24	6.76E-02	3.77E-01	0.06
Creatine	0.02	5.29E-01	6.54E-01	0.05	-0.21	1.01E-01	4.04E-01	0.02
Creatinine	0.27	5.79E-01	6.79E-01	0.31	-0.37	2.03E-01	5.31E-01	0.27
Cysteine	0.23	3.53E-01	6.22E-01	0.56	0.24	1.22E-01	4.15E-01	0.23
Cystine	0.09	4.81E-01	6.54E-01	0.70	-0.04	8.97E-01	9.68E-01	0.09
Docosahexaenoic acid	0.40	5.24E-02	3.41E-01	1.80	-0.05	6.96E-01	9.68E-01	0.40
Docosapentaenoic acid	0.00	9.71E-01	1.00E+00	0.02	0.36	6.76E-02	3.77E-01	0.00
Eicosanoic acid	0.23	1.23E-01	4.64E-01	1.33	0.18	4.08E-01	8.41E-01	0.23
Eicosapentaenoic acid	0.18	3.93E-01	6.22E-01	0.82	-0.18	8.97E-01	9.68E-01	0.18
Eicosenoic acid	0.13	1.05E-01	4.20E-01	1.00	0.45	8.55E-03	1.94E-01	0.13
Erucic acid	-0.27	5.20E-03	1.77E-01	2.08	-0.29	1.46E-01	4.50E-01	-0.27
g-Linolenic acid	-0.20	7.53E-02	3.41E-01	1.39	-0.01	8.29E-01	9.68E-01	-0.20
Glutamic acid	0.07	5.29E-01	6.54E-01	0.66	-0.23	1.22E-01	4.15E-01	0.07
Glutamine	-0.33	1.85E-02	2.52E-01	1.81	-0.25	8.29E-01	9.68E-01	-0.33
GSH	-0.09	4.81E-01	6.54E-01	0.60	-0.21	2.66E-02	3.02E-01	-0.09
Glycine	-0.47	3.89E-03	1.77E-01	1.95	-0.78	8.29E-01	9.68E-01	-0.47
GSSG	0.25	1.15E-02	2.52E-01	1.51	0.02	9.65E-01	9.80E-01	0.25
Histidine	0.08	7.53E-02	3.41E-01	1.54	-0.03	4.60E-01	8.93E-01	0.08
Homocysteine	0.10	2.18E-01	5.48E-01	0.55	0.04	8.29E-01	9.68E-01	0.10
Isoleucine	0.08	2.80E-01	6.12E-01	0.41	0.09	7.62E-01	9.68E-01	0.08
Leucine	-0.10	3.15E-01	6.12E-01	0.87	-0.12	8.29E-01	9.68E-01	-0.10
Linoleic acid	0.09	4.36E-01	6.54E-01	0.47	-0.04	7.62E-01	9.68E-01	0.09
Lysine	0.18	3.53E-01	6.22E-01	0.58	0.11	5.15E-01	9.21E-01	0.18
Methionine	0.09	3.15E-01	6.12E-01	0.87	0.22	4.34E-02	3.69E-01	0.09
Nervonic acid	0.22	7.53E-02	3.41E-01	1.33	0.51	4.39E-03	1.94E-01	0.22
Octadecanoic acid	0.01	1.00E+00	1.00E+00	0.12	-0.28	8.97E-01	9.68E-01	0.01
Oleic acid	0.01	3.15E-01	6.12E-01	0.26	0.24	8.31E-02	3.77E-01	0.01
Ornithine	0.02	9.71E-01	1.00E+00	0.25	-0.05	9.65E-01	9.80E-01	0.02
Palmitic acid	-0.24	5.29E-01	6.54E-01	0.39	-0.48	4.60E-01	8.93E-01	-0.24
Palmitoleic acid	0.08	1.65E-01	4.89E-01	0.44	0.19	1.46E-01	4.50E-01	0.08
Phenylalanine	0.14	3.93E-01	6.22E-01	0.52	-0.21	2.03E-01	5.31E-01	0.14
Proline	-0.16	9.12E-01	9.84E-01	0.34	-0.10	6.96E-01	9.68E-01	-0.16
Pyroglutamic acid	0.07	7.39E-01	8.38E-01	0.35	-0.20	3.43E-02	3.33E-01	0.07
Serine	-0.41	1.90E-01	4.98E-01	1.04	-0.28	8.29E-01	9.68E-01	-0.41
Tetradecanoic acid	-0.03	7.96E-01	8.87E-01	0.28	-0.08	3.15E-01	6.92E-01	-0.03
Threonine	-0.01	6.84E-01	7.89E-01	0.17	-0.06	8.97E-01	9.68E-01	-0.01
Tryptophan	-0.01	1.00E+00	1.00E+00	0.15	0.11	3.15E-01	6.92E-01	-0.01
Tyrosine	0.06	3.93E-01	6.22E-01	0.27	0.05	5.73E-01	9.68E-01	0.06
Valine	0.47	4.33E-02	3.41E-01	1.99	0.185	1.22E-01	4.15E-01	0.47

Supplementary Table S5. Univariate ROC curve analysis in plasma				
Rank	Potential biomarkers	AUC	P-values	Log2FC
COPD1/VC1				
1	Malonic acid/3-Methylhistidine	0.99	3.7E-07	-5.83
2	Malonic acid/Docosapentaenoic acid	0.97	1.6E-05	-0.41
3	Linoleic acid/Docosapentaenoic acid	0.97	8.2E-05	-0.14
4	Glutamic acid/3-Methylhistidine	0.97	3.4E-05	-0.18
5	a-Aminobutyric acid/Histidine	0.97	1.9E-04	-0.20
6	Malonic acid/Oxaloacetic acid	0.96	5.8E-05	-0.16
7	g-Linolenic acid/Docosapentaenoic acid	0.96	2.9E-04	-0.83
8	Glutamic acid/Histidine	0.96	4.5E-05	-4.47
9	Malonic acid/Histidine	0.95	9.7E-05	-0.15
10	g-Linolenic acid/Docosahexaenoic acid	0.95	3.0E-04	-0.10
11	2-Hydroxybutyric acid/Histidine	0.94	1.2E-04	-0.45
12	g-Linolenic acid/Homocysteine	0.94	1.9E-04	-0.24
13	2-Hydroxybutyric acid/3-Methylhistidine	0.93	6.6E-05	-0.39
14	Fumaric acid/Docosapentaenoic acid	0.93	2.0E-04	-4.96
15	Malonic acid/Erucic acid	0.92	3.3E-04	-1.55
16	4-Hydroxyphenyllactic acid/Histidine	0.92	2.8E-04	-0.17
17	a-Ketoglutaric acid/Docosapentaenoic acid	0.91	2.5E-04	-0.30
18	Phenylalanine/3-Methylhistidine	0.91	1.5E-04	-0.09
19	4-Hydroxyphenyllactic acid/3-Methylhistidine	0.9	2.2E-04	-1.31
20	GSSG	0.87	5.8E-03	-0.09
21	g-Linolenic acid	0.86	2.8E-03	-0.13
22	Histidine	0.83	4.2E-02	0.05
23	Glutathione	0.81	1.2E-02	-0.13
24	a-Aminobutyric acid	0.81	2.7E-02	-0.12
25	Malonic acid	0.78	3.0E-02	-0.27
26	3-Methylhistidine	0.77	4.7E-03	0.38
27	Docosapentaenoic acid	0.76	1.3E-02	0.13
28	Pyruvic acid	0.75	1.2E-01	-0.08
29	Succinic acid	0.74	2.8E-02	-0.09
30	Fumaric acid	0.74	8.2E-02	-0.11
31	Octadecanoic acid	0.74	7.8E-02	0.04
32	Homocysteine	0.74	3.8E-02	0.13
33	Glutamic acid	0.74	6.4E-02	-0.04
34	1-Methylhistidine	0.74	5.1E-02	0.10
35	2-Hydroxybutyric acid	0.73	5.7E-02	-0.12
36	Erucic acid	0.72	1.9E-01	0.08
37	Eicosapentaenoic acid	0.71	7.7E-02	0.06
38	Glycolic acid	0.7	8.1E-02	-0.02
39	a-Ketoglutaric acid	0.7	1.1E-01	-0.04
40	4-Hydroxyphenyllactic acid	0.7	1.0E-01	-0.14
COPD2/VC2				
1	Phenylalanine/a-Aminobutyric acid	1.00	2.7E-06	0.15
2	Phenylalanine/Glutamine	1.00	2.4E-05	0.20
3	Valine/a-Aminobutyric acid	1.00	6.8E-05	0.09
4	Phenylalanine/Alanine	0.99	3.2E-05	0.66
5	Tyrosine/Alanine	0.99	6.0E-05	1.49
6	4-Hydroxyphenylacetic acid/a-Aminobutyric acid	0.98	6.9E-05	0.32
7	4-Hydroxyphenylacetic acid/Alanine	0.98	2.2E-05	0.15
8	4-Hydroxyphenylacetic acid/Serine	0.98	1.6E-04	0.14
9	4-Hydroxyphenylacetic acid/Glutamine	0.98	6.8E-05	0.10
10	Leucine/a-Aminobutyric acid	0.98	2.8E-04	0.11
11	a-Aminobutyric acid/histidine	0.98	2.3E-04	-0.11
12	Alanine/Citrulline	0.98	4.8E-05	-0.48
13	Glutamine/Histidine	0.98	1.5E-04	-0.09
14	4-Hydroxyphenylacetic acid/Proline	0.96	2.3E-04	0.16
15	Erucic acid/Alanine	0.96	3.2E-04	0.17
16	Pyruvic acid/Erucic acid	0.95	2.4E-04	-0.16
17	Valine/Alanine	0.95	1.9E-04	0.74
18	Creatine/Citrulline	0.95	3.2E-04	-0.34
19	4-Hydroxy-L-proline/Alanine	0.94	2.3E-04	0.20
20	Octadecanoic acid	0.89	7.7E-03	0.11
21	4-Hydroxyphenylacetic acid	0.88	8.3E-03	2.07
22	Erucic acid	0.86	3.9E-03	0.55
23	Pyruvic acid	0.85	1.8E-02	-0.04
24	Alanine	0.83	7.3E-03	-0.07
25	Glycine	0.81	3.3E-02	-0.04
26	Serine	0.80	3.8E-02	-0.04

27	Nervonic acid	0.79	2.5E-02	0.57
28	2-Hydroxyglutaric acid	0.76	4.9E-02	-0.09
29	Eicosanoic acid	0.76	1.5E-01	0.24
30	Creatine	0.76	4.0E-02	-0.04
31	a-Ketoglutaric acid	0.75	6.3E-02	0.05
32	Arachidonic acid	0.75	5.5E-02	0.05
33	a-Aminobutyric acid	0.75	5.9E-02	-0.10
34	Ornithine	0.75	5.5E-02	0.05
35	Creatinine	0.74	1.1E-01	-0.07
36	Citrulline	0.74	6.5E-02	0.04
37	Glutamine	0.73	9.1E-02	-0.03
38	Cystine	0.73	1.0E-01	0.06
39	3-Methylhistidine	0.73	1.2E-01	0.19
40	g-Linolenic acid	0.71	2.3E-01	-0.11
41	Phenylalanine	0.71	1.7E-01	0.04
42	4-Hydroxy-L-proline	0.70	1.5E-01	0.06
43	Aspartic acid	0.70	2.2E-01	-0.05

Supplementary Table S6. Statistical results of determined metabolites in the urine between COPD1 and VC1 or COPD2 and VC2.

Name	log2FC(COPD1/ VC1)	p-value of COPD1 and VC1	FDR of COPD1 and VC1	VIP in PLS-DA of COPD1 and VC1	log2FC(COPD2/ VC2)	p-value of COPD2 and VC2	FDR of COPD2 and VC2	VIP in PLS-DA of COPD2 and VC2
1-Methylhistidine	0.34	1.23E-01	2.38E-01	0.74	-1.09	1.47E-02	9.10E-02	1.17
2-Hydroxybutyric acid	0.16	1.90E-01	2.79E-01	0.77	0.30	1.85E-02	9.10E-02	1.21
2-Hydroxyglutaric acid	0.20	2.47E-01	3.23E-01	0.64	0.11	1.90E-01	2.38E-01	0.44
3-Methylhistidine	0.40	1.23E-01	2.38E-01	0.73	-0.98	2.88E-02	9.10E-02	1.15
4-Hydroxyhippuric acid	0.43	1.23E-01	2.38E-01	1.06	-0.46	1.05E-01	1.58E-01	0.76
4-Hydroxy-L-proline	0.34	6.30E-02	1.80E-01	0.73	-0.75	7.53E-02	1.19E-01	1.06
4-Hydroxyphenylacetic acid	-0.51	1.85E-02	1.11E-01	1.31	0.25	3.15E-01	3.63E-01	0.58
4-Hydroxyphenyllactic acid	-0.28	1.43E-01	2.39E-01	1.08	0.09	3.93E-01	4.29E-01	0.47
a-Ketoglutaric acid	0.77	1.47E-02	1.10E-01	1.38	0.01	6.31E-01	6.52E-01	0.16
Alanine	0.63	1.85E-02	1.11E-01	1.33	-0.51	1.23E-01	1.72E-01	0.88
Alpha-aminoadipic acid	0.77	4.33E-02	1.62E-01	0.93	-1.91	2.32E-02	9.10E-02	1.28
Arachidonic acid	0.50	4.87E-04	1.46E-02	1.82	0.01	6.84E-01	6.84E-01	0.11
Arginine	-0.16	4.36E-01	5.13E-01	0.02	-0.65	1.47E-02	9.10E-02	1.25
Asparagine	0.49	6.30E-02	1.80E-01	1.07	-0.89	4.33E-02	9.53E-02	1.12
Aspartic acid	0.31	8.92E-02	2.06E-01	1.18	-0.43	1.85E-02	9.10E-02	1.26
b-Alanine	0.21	3.15E-01	3.86E-01	0.65	-0.28	2.18E-01	2.61E-01	0.50
b-Aminobutyric acid	0.09	6.31E-01	6.76E-01	0.32	0.10	5.29E-01	5.57E-01	0.07
cis-Aconitic acid	0.00	7.39E-01	7.65E-01	0.08	0.21	1.43E-01	1.83E-01	0.86
Citric acid	0.29	1.43E-01	2.39E-01	1.02	0.12	1.43E-01	1.83E-01	0.52
Citrulline	0.84	3.55E-02	1.42E-01	1.05	-1.05	5.24E-02	9.53E-02	0.98
Creatine	0.14	4.81E-01	5.45E-01	0.28	-0.97	4.33E-02	9.53E-02	0.96
Creatinine	0.52	2.88E-02	1.23E-01	1.27	-0.36	1.23E-01	1.72E-01	0.76
Cysteine	0.58	2.32E-02	1.16E-01	0.95	-0.33	3.53E-01	3.92E-01	0.46
Cystine	0.44	1.65E-01	2.55E-01	0.92	-0.39	1.50E-03	6.27E-02	1.55
Docosahexaenoic acid	0.24	1.65E-01	2.55E-01	0.90	0.59	3.55E-02	9.53E-02	1.04
Eicosenoic acid	-0.27	2.18E-01	2.90E-01	0.68	0.29	1.43E-01	1.83E-01	0.72
Fumaric acid	0.81	1.05E-03	2.10E-02	1.83	0.12	3.53E-01	3.92E-01	0.36
Gamma-aminobutyric acid	0.40	2.32E-02	1.16E-01	1.34	-0.11	2.18E-01	2.61E-01	0.37
Glutamic acid	0.02	4.81E-01	5.45E-01	0.28	-0.67	2.32E-02	9.10E-02	1.23
Glutamine	0.81	8.93E-03	8.93E-02	1.51	-0.71	2.88E-02	9.10E-02	1.05
Glycine	0.50	6.30E-02	1.80E-01	1.13	-0.61	2.88E-02	9.10E-02	1.12
Glycolic acid	-0.05	6.84E-01	7.20E-01	0.34	0.25	3.55E-02	9.53E-02	1.16
Hippuric acid	0.79	8.93E-03	8.93E-02	1.52	-0.77	3.55E-02	9.53E-02	1.25
Histidine	0.46	1.90E-01	2.79E-01	0.88	-0.84	7.53E-02	1.19E-01	1.06
Homocystine	0.31	5.20E-03	7.79E-02	1.62	-0.30	1.47E-02	9.10E-02	1.26
Homoserine	0.51	2.88E-02	1.23E-01	1.23	-0.92	2.88E-02	9.10E-02	1.29
Isocitric acid	0.19	8.92E-02	2.06E-01	0.88	0.49	6.84E-03	9.10E-02	1.25
Isoleucine	0.32	1.23E-01	2.38E-01	1.01	-0.62	1.15E-02	9.10E-02	1.26
Lactic acid	0.23	2.18E-01	2.90E-01	1.06	0.30	2.88E-02	9.10E-02	1.09
Leucine	0.12	7.96E-01	8.09E-01	0.29	-0.50	6.30E-02	1.05E-01	0.99
Linoleic acid	-0.66	8.92E-02	2.06E-01	0.82	-0.09	6.84E-01	6.84E-01	0.03
L-Targinine	0.13	1.00E+00	1.00E+00	0.11	-1.17	5.24E-02	9.53E-02	1.20
Lysine	0.29	2.18E-01	2.90E-01	0.61	-0.73	1.23E-01	1.72E-01	0.99
Malic acid	0.65	3.25E-04	1.46E-02	1.87	0.10	2.47E-01	2.91E-01	0.41
Malonic acid	-0.15	3.15E-01	3.86E-01	0.76	0.11	5.29E-01	5.57E-01	0.44
Methionine	0.29	1.43E-01	2.39E-01	0.64	-1.31	1.47E-02	9.10E-02	1.33
N-Methyl-D-aspartic acid	0.73	1.47E-02	1.10E-01	1.37	-0.77	4.33E-02	9.53E-02	1.07
Ornithine	0.24	5.24E-02	1.80E-01	1.04	-0.36	1.43E-01	1.83E-01	0.90
Oxaloacetic acid	-0.09	5.29E-01	5.88E-01	0.45	0.73	2.09E-03	6.27E-02	1.46
Phenylalanine	0.26	2.18E-01	2.90E-01	0.73	-0.33	5.24E-02	9.53E-02	0.91
Pipecolic acid	0.63	6.30E-02	1.80E-01	1.16	-0.90	4.33E-02	9.53E-02	1.13
Proline	0.38	1.43E-01	2.39E-01	0.97	-0.97	3.89E-03	7.77E-02	1.47
Pyruvic acid	0.14	4.36E-01	5.13E-01	0.56	0.37	5.24E-02	9.53E-02	1.04
Sarcosine	0.31	1.65E-01	2.55E-01	0.60	-1.07	5.24E-02	9.53E-02	1.27
Serine	0.38	7.53E-02	1.96E-01	0.95	-0.57	5.24E-02	9.53E-02	1.07
Succinic acid	0.13	5.79E-01	6.31E-01	0.44	0.25	6.30E-02	1.05E-01	0.96
Threonine	0.58	7.53E-02	1.96E-01	1.04	-0.87	5.24E-02	9.53E-02	1.09
Tryptophan	0.29	1.05E-01	2.34E-01	0.93	-0.25	6.30E-02	1.05E-01	0.80
Tyrosine	0.18	2.80E-01	3.57E-01	0.56	-0.47	8.92E-02	1.37E-01	0.97
Valine	0.42	1.43E-01	2.39E-01	0.95	-0.48	2.88E-02	9.10E-02	1.18

**Supplementary Table S7.** Univariate ROC curve analysis in urine				
Rank	Potential biomarkers	AUC	P-values	Log2FC
COPD1/VC1				
1	Succinic acid/Malic acid	1.00	5.4E-06	-0.29
2	Succinic acid/Fumaric acid	0.98	8.1E-05	-0.21
3	Glutamine/b-aminobutyric acid	0.98	1.1E-04	0.33
4	Malic acid/cis-Aconitic acid	0.97	2.2E-05	0.47
5	Malic acid/2-Hydroxyglutaric acid	0.96	7.9E-05	0.91
6	Malic acid/Linoleic acid	0.96	8.6E-05	0.14
7	Oxaloacetic acid/Malic acid	0.95	6.5E-05	-0.31
8	Linoleic acid/Arachidonic acid	0.95	1.8E-04	-0.09
9	N-Methyl-D-aspartic acid/b-Alanine	0.95	1.4E-04	0.17
10	Malonic acid/Malic acid	0.94	1.9E-04	-0.47
11	Threonine/L-Targinine	0.94	8.5E-05	0.20
12	Citrulline/L-Targinine	0.94	1.5E-04	0.31
13	Fumaric acid/Oxaloacetic acid	0.93	2.5E-04	1.17
14	Fumaric acid/cis-Aconitic acid	0.93	2.1E-04	0.28
15	Fumaric acid/Linoleic acid	0.93	1.7E-04	0.25
16	Glycolic acid/Malic acid	0.93	1.6E-04	-4.25
17	Malic acid/4-Hydroxyphenyllactic acid	0.92	5.9E-05	0.31
18	Fumaric acid/4-Hydroxyphenyllactic acid	0.91	1.9E-04	0.72
19	4-Hydroxyphenyllactic acid/Arachidonic acid	0.91	1.3E-04	-0.18
20	Malic acid	0.90	6.8E-04	2.01
21	Fumaric acid	0.88	1.1E-03	0.36
22	Arachidonic acid	0.87	2.1E-03	0.38
23	4-Hydroxyphenylacetic acid	0.86	8.3E-03	-0.20
24	Hippuric acid	0.83	1.0E-02	0.08
25	Glutamine	0.82	1.1E-02	0.11
26	a-Ketoglutaric acid	0.81	2.6E-02	0.54
27	Homocysteine	0.79	1.6E-02	0.14
28	N-Methyl-D-aspartic acid	0.79	2.8E-02	0.45
29	Alanine	0.78	3.7E-02	0.11
30	4-Hydroxyphenyllactic acid	0.77	2.1E-02	-0.11
31	Citrulline	0.77	1.1E-01	0.14
32	Cysteine	0.77	1.6E-01	0.09
33	Homoserine	0.75	6.3E-02	0.10
34	a-Aminoadipic acid	0.74	1.6E-01	0.31
35	Threonine	0.74	1.3E-01	0.10
36	Creatinine	0.74	5.5E-02	0.06
37	g-Aminobutyric acid	0.74	4.8E-02	0.07
38	Malonic acid	0.72	5.2E-02	-0.11
39	Linoleic acid	0.72	7.5E-02	-0.02
40	Glycine	0.72	1.0E-01	0.07
41	Asparagine	0.72	1.2E-01	0.09
42	4-Hydroxyhippuric acid	0.71	1.2E-01	0.07
43	Pipecolic acid	0.71	7.4E-02	0.11
44	Eicosenoic acid	0.70	1.2E-01	0.00
COPD2/VC2				
1	Glycine/b-Aminobutyric acid	0.98	1.7E-05	-0.51
2	2-Hydroxybutyric acid/Cystine	0.96	2.2E-04	0.07
3	Lactic acid/Hippuric acid	0.95	1.9E-04	0.10
4	Lactic acid/Homocystine	0.95	2.4E-04	0.23
5	Lactic acid/Cystine	0.95	2.5E-04	0.14
6	Oxaloacetic acid/Cystine	0.95	2.6E-04	0.16
7	Lactic acid/Homoserine	0.93	1.4E-04	0.21
8	Valine/b-Aminobutyric acid	0.93	2.1E-04	-0.83
9	Pipecolic acid/b-Alanine	0.93	1.7E-04	-0.72
10	Threonine/b-Alanine	0.93	2.0E-04	-2.49
11	Isocitric acid/Glutamic acid	0.92	4.1E-04	0.26
12	Isocitric acid/Arginine	0.92	1.9E-04	0.25
13	leucine/b-Aminobutyric acid	0.92	2.1E-04	-4.83
14	N-Methyl-D-aspartic acid/b-Alanine	0.92	3.3E-04	-0.16
15	Cystine	0.91	5.3E-04	-0.11
16	Lactic acid/Sarcosine	0.91	2.7E-04	0.26
17	Tryptophan/proline	0.91	2.1E-04	5.17
18	Docosahexaenoic acid/Proline	0.90	3.3E-04	0.23
19	Phenylalanine/Proline	0.90	1.9E-04	0.51
20	b-Alanine/Histidine	0.90	2.6E-04	3.71
21	Proline	0.89	1.1E-03	-0.42
22	Arginine	0.88	4.9E-03	-0.12

23	Homocystine	0.87	1.6E-03	-0.23
24	Oxaloacetic acid	0.86	7.5E-03	0.23
25	Isoleucine	0.86	5.5E-03	-0.17
26	Glutamic acid	0.86	6.2E-03	-0.13
27	Aspartic acid	0.84	3.5E-03	-0.12
28	Isocitric acid	0.83	2.2E-02	0.47
29	1-Methylhistidine	0.83	1.7E-02	-0.25
30	Hippuric acid	0.82	1.1E-02	-0.09
31	Methionine	0.82	6.0E-03	-0.24
32	Homoserine	0.82	6.3E-03	-0.20
33	Valine	0.81	8.2E-03	-0.18
34	Glutamine	0.81	2.6E-02	-0.11
35	a-Aminoadipic acid	0.80	9.2E-03	-0.68
36	Pipecolic acid	0.80	1.9E-02	-0.16
37	N-Methyl-D-aspartic acid	0.80	2.5E-02	-0.43
38	Glycine	0.80	1.8E-02	-0.14
39	Creatine	0.80	5.7E-02	-0.17
40	3-Methylhistidine	0.80	1.8E-02	-0.19
41	Threonine	0.79	2.3E-02	-0.17
42	Sarcosine	0.79	8.0E-03	-0.26
43	Asparagine	0.79	2.1E-02	-0.18
44	Serine	0.78	2.0E-02	-0.14
45	Citrulline	0.78	4.6E-02	-0.20
46	L-Targinine	0.78	1.3E-02	-0.41
47	Histidine	0.77	2.8E-02	-0.17
48	Phenylalanine	0.76	4.1E-02	-0.14
49	Tyrosine	0.76	3.3E-02	-0.12
50	Leucine	0.76	3.3E-02	-0.15
51	Docosahexaenoic acid	0.75	5.9E-02	0.11
52	Tryptophan	0.75	6.4E-02	-0.16
53	4-Hydroxy-L-proline	0.75	2.7E-02	-0.17
54	Ornithine	0.75	4.2E-02	-0.08
55	4-Hydroxyhippuric acid	0.74	1.0E-01	-0.07
56	Creatinine	0.74	9.8E-02	-0.06
57	Lysine	0.74	3.9E-02	-0.14
58	Pyruvic acid	0.73	1.0E-01	0.17
59	Lactic acid	0.72	7.7E-02	0.23
60	Glycolic acid	0.72	1.0E-01	0.55
61	Alanine	0.72	6.3E-02	-0.14
62	2-Hydroxybutyric acid	0.70	6.9E-02	0.03
63	b-Alanine	0.70	2.7E-01	-0.07

Supplementary Table S8. Selected reaction monitoring conditions for 45 organic metabolites include amino acids and IS profiling analysis by LC-MS/MS

No	Metabolites	Electro spray ionization mode	SRM (*m/z*)	CE (V)
1	Hydroxyhippuric acid	+	195.90>121.15	-13
2	Hippuric acid	+	180.20>105.15	-13
3	Pyroglutamic acid	+	130.00>84.20	-13
4	Tryptophan	+	205.10>188.00	-10
5	Phenylalanine	+	166.10>120.00	-15
6	Tyrosine	+	182.10>136.10	-12
7	Leucine	+	132.00>43.20	-23
8	Methionine	+	150.00>56.10	-16
9	Isoleucine	+	132.10>69.20	-16
10	Homocysteine	+	135.80>90.10	-10
11	Valine	+	118.10>72.10	-10
12	Cysteine	+	122.00>59.10	-22
13	GSH	+	307.80>179.15	-12
14	a-Aminoadipic acid	+	161.90>98.10	-14
15	Pipecolic acid	+	130.10>84.10	-15
16	Glutamic acid	+	148.00>84.00	-15
17	Proline	+	116.10>70.00	-15
18	Threonine	+	120.00>74.20	-11
19	4-Hydroxyproline	+	132.00>86.10	-14
20	Aspartic acid	+	134.00>74.15	-13
21	Alanine	+	90.00>44.15	-11
22	Serine	+	106.00>60.15	-11
23	Glutamine	+	147.00>84.20	-16
24	Glycine	+	75.80>30.15	-12
25	Creatine	+	132.20>90.15	-13
26	Asparagine	+	133.00>74.15	-15
27	Creatinine	+	114.00>86.15	-13
28	Citrulline	+	176.00>70.20	-22
29	b-Aminoisobutyric acid	+	104.00>86.15	-10
30	b-Alanine	+	89.90>72.20	-11
31	g-Aminobutyric acid	+	104.00>87.10	-11
32	GSSG	+	613.00>484.10	-16
33	1-Methylhistidine	+	169.90>124.15	-14
34	Histidine	+	156.00>110.00	-15
35	3-Methylhistidine	+	169.90>95.20	-30
36	NG-Methylarginine	+	189.30>70.20	-23
37	Lysine	+	147.10>84.10	-15
38	γ-Hydroxylysine	+	162.90>128.20	-13
39	Ornithine	+	132.90>70.10	-17
40	Arginine	+	175.10>70.10	-25
41	Sarcosine	+	89.90>44.15	-13
42	N-Methyl-DL-aspartic acid	+	148.00>87.95	-10
43	Homoserine	+	120.10>74.15	-12
44	a-Aminobutyric acid	+	104.00>58.25	-11
45	5-Hydroxytryptophan	+	220.90>204.25	-10
IS	$^{13}C_1$-Phenylalanine	+	167.10>120.10	-15

Supplementary Table S9. Selected reaction monitoring conditions for the 24 fatty acid, 18 organic acid and 3 ISs profiling analysis by GC-MS/MS

No	Metabolites	Ionization mode	SRM (*m/z*)	CE (V)
1	Pyruvic acid	EI	174.00>74.10	15
2	Lactic acid	EI	261.00>147.10	15
3	Glycolic acid	EI	247.00>147.10	15
4	2-Hydroxybutyric acid	EI	275.00>147.10	15
5	3-Hydroxybutyric acid	EI	275.00>159.20	5
6	Malonic acid	EI	275.00>73.10	20
7	Succinic acid	EI	289.00>147.10	10
8	Fumaric acid	EI	287.00>147.10	15
9	Oxaloacetic acid	EI	332.00>147.10	10
10	a-Ketoglutaric acid	EI	346.00>156.10	10
11	C14:0	EI	285.00>131.10	10
12	4-Hydroxyphenylacetic acid	EI	323.00>205.10	10
13	Malic acid	EI	419.00>115.10	10
14	2-Hydroxyglutaric acid	EI	433.00>245.20	15
15	C16:1	EI	311.00>131.10	10
16	C16:0	EI	313.00>131.10	10
17	cis-Aconitic acid	EI	459.00>147.10	20
18	g-C18:3	EI	335.00>243.20	5
19	C18:2	EI	337.00>131.20	15
20	C18:1	EI	339.00>131.10	10
21	a-C18:3	EI	335.00>75.00	30
22	C18:0	EI	341.00>131.10	10
23	4-Hydroxyphenyllactic acid	EI	467.00>439.30	5
24	Citric acid	EI	459.00>147.10	20
25	Isocitric acid	EI	459.00>147.20	20
26	C20:4	EI	361.00>269.00	5
27	C20:5	EI	359.00>75.00	30
28	C20:1	EI	367.00>131.20	10
29	C20:0	EI	369.00>131.10	15
30	C22:6	EI	385.00>75.10	24
31	C22:5	EI	387.00>75.20	20
32	C22:1	EI	395.00>131.10	15
33	C24:1	EI	423.00>131.10	15
34	Acetoacetic acid	EI	188.00>89.10	9
35	C6:0	EI	173.00>131.10	5
36	C8:0	EI	201.00>131.10	5
37	C10:0	EI	229.00>131.10	5
38	C12:0	EI	257.00>131.10	10
39	C14:1	EI	283.00>131.10	10
40	C22:0	EI	397.00>131.10	10
41	C24:0	EI	425.00>131.20	10
42	C26:0	EI	453.00>131.20	15
IS[1]	$^{13}C_2$- Succinic acid	EI	291.00>147.10	10
IS[2]	3,4-Dimethoxybenzoic acid	EI	239.00>195.10	10
IS[3]	C15:0	EI	299.00>131.10	10

Supplementary Figures

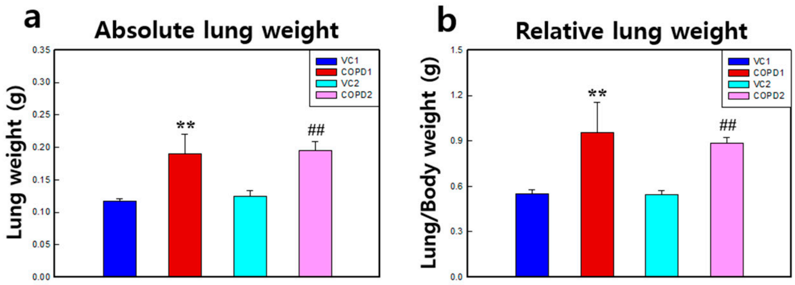

Supplementary Figure S1. Absolute lung weight (a) and relative lung weight, of the four groups (VC1, COPD1, VC2, and COPD2). Data are presented as mean ± SD (n = 10), ** $P < 0.01$, compared with the VC1, ## $P < 0.01$, compared with the VC2.

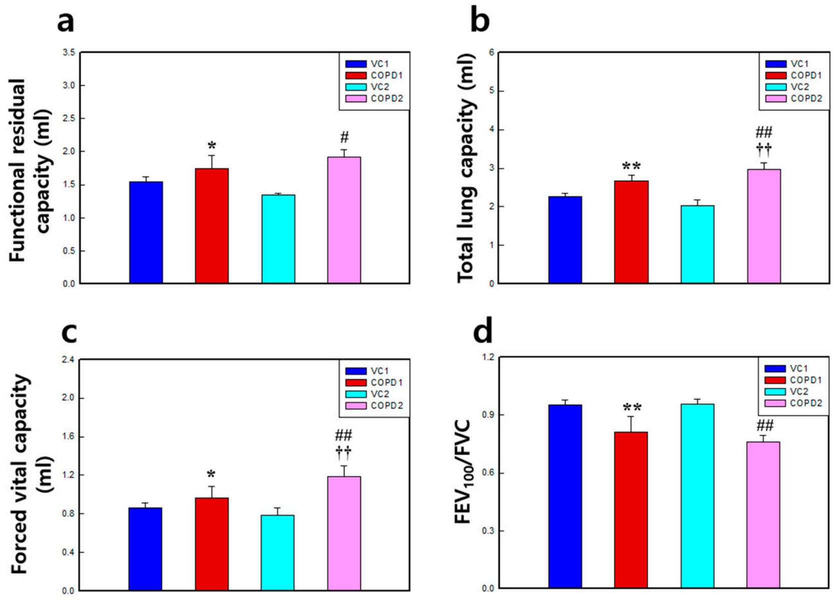

Supplementary Figure S2. Lung function measurement, including FRC (a), TLC (b), FVC (c), and FEV_{100}/FVC, of the four groups (VC1, COPD1, VC2, and COPD2). Data are presented as mean ± SD (n = 10), * $P < 0.05$, ** P < 0.01, compared with the VC1, $^{\#}$ $P < 0.05$, $^{\#\#}$ $P < 0.01$, compared with the VC2, †† $P < 0.01$, compared with the COPD1.

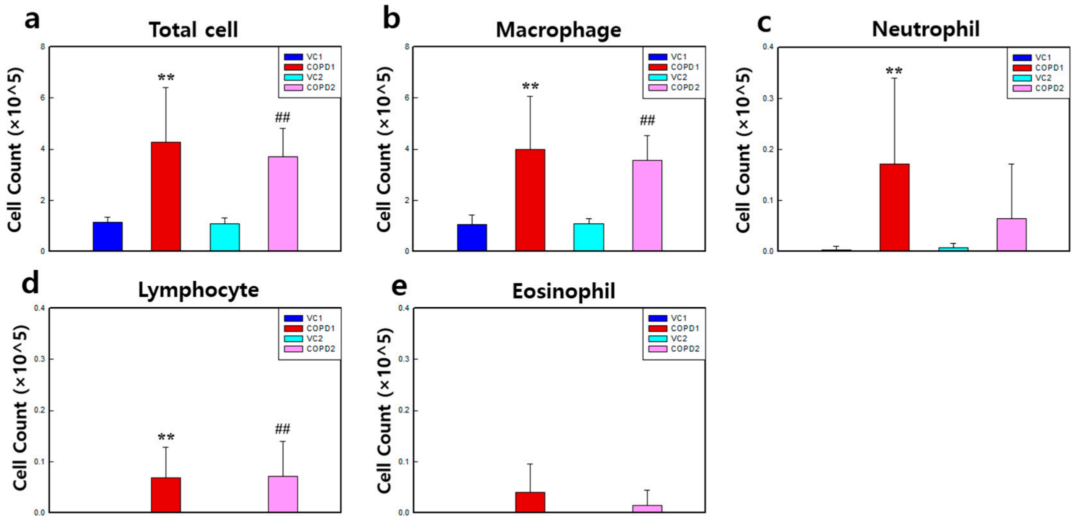

Supplementary Figure S3. The number of total cell (a), macrophage (b), neutrophil (c), lymphocyte (d), and eosinophil (e) in BALF, of the four groups (VC1, COPD1, VC2, and COPD2). Data are presented as mean ± SD (n = 10), ** $P < 0.01$, compared with the VC1, $^{\#\#}$ $P < 0.01$, compared with the VC2.

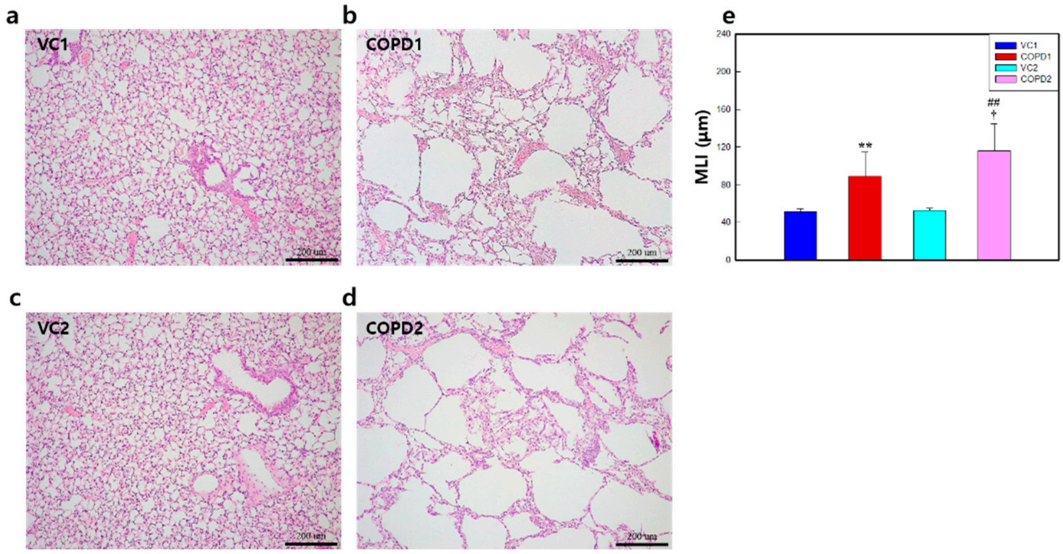

Supplementary Figure S4. Representative light micrographs of lung alveolar spaces in H&E-stained sections of VC1 (a), COPD1 (b), VC2 (c), and COPD2 (d). Scale bars = 200 μm. The mean values of MLI (e), of the four groups (VC1, COPD1, VC2, and COPD2). Data are presented as mean ± SD (n = 10), ** $P < 0.01$, compared with the VC1, $^{\#\#}$ $P < 0.01$, compared with the VC2, † $P < 0.05$, compared with the COPD1.

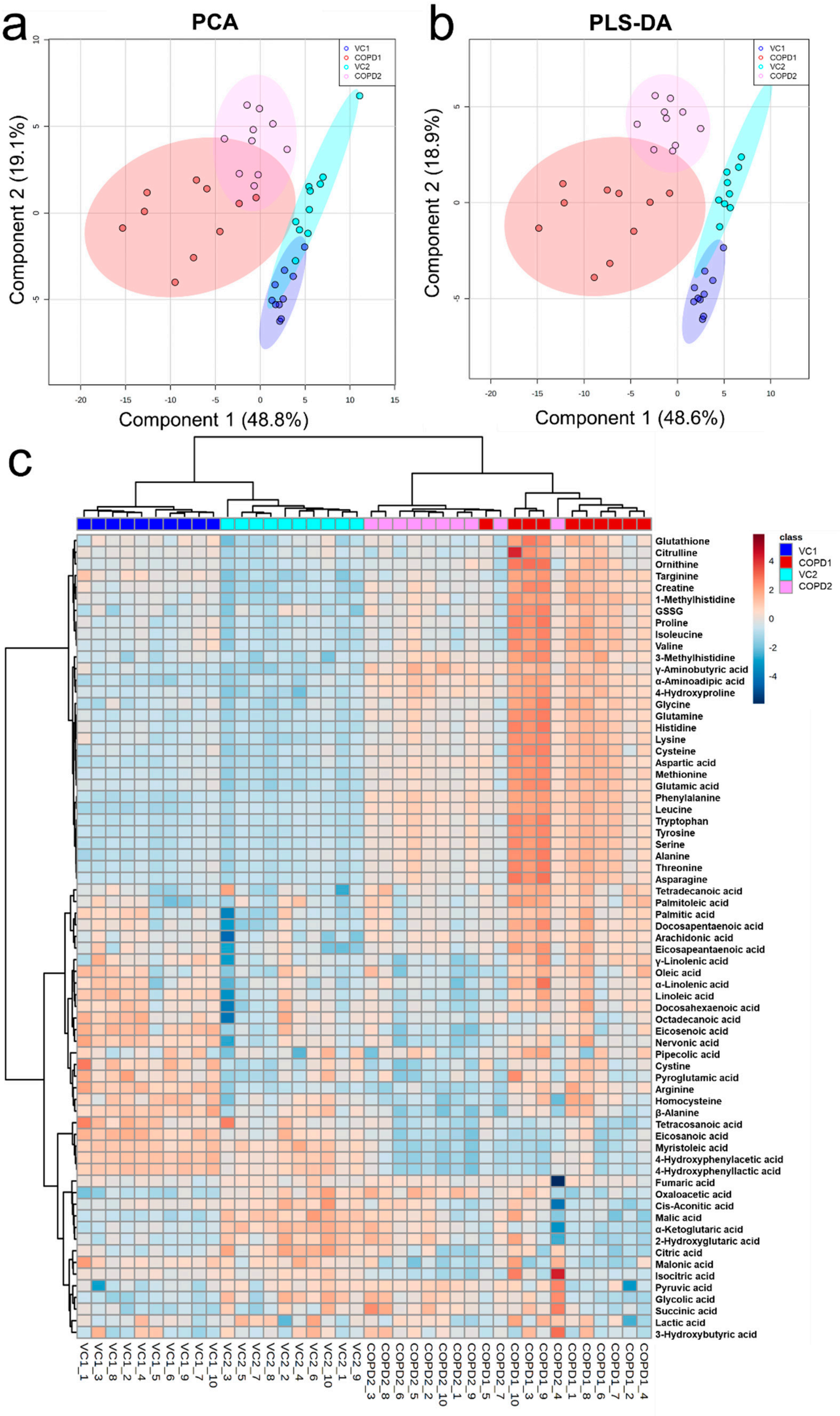

Supplementary Figure S5. PCA scores plot (a), PLS-DA scores plot (b), and hierarchical clustering heatmap (c) of the lung in the four groups (COPD1, VC1, COPD2, and VC2).

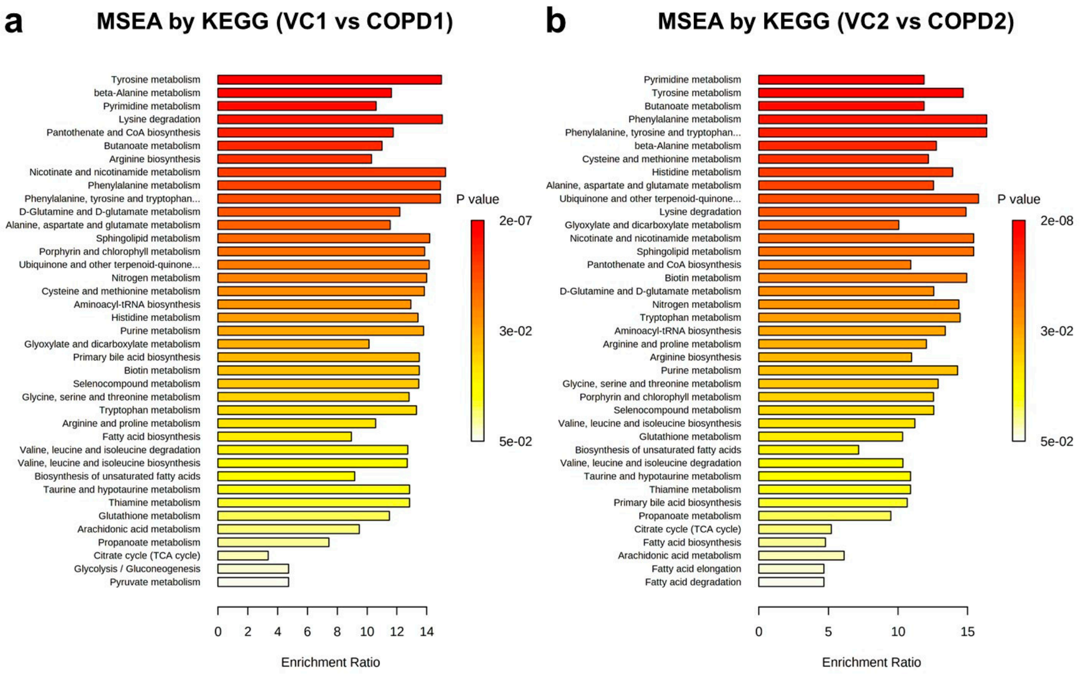

Supplementary Figure S6. Quantitative metabolite set enrichment analysis (MSEA) of lung in COPD1 and VC1 (a), and COPD2 and VC2 (b).

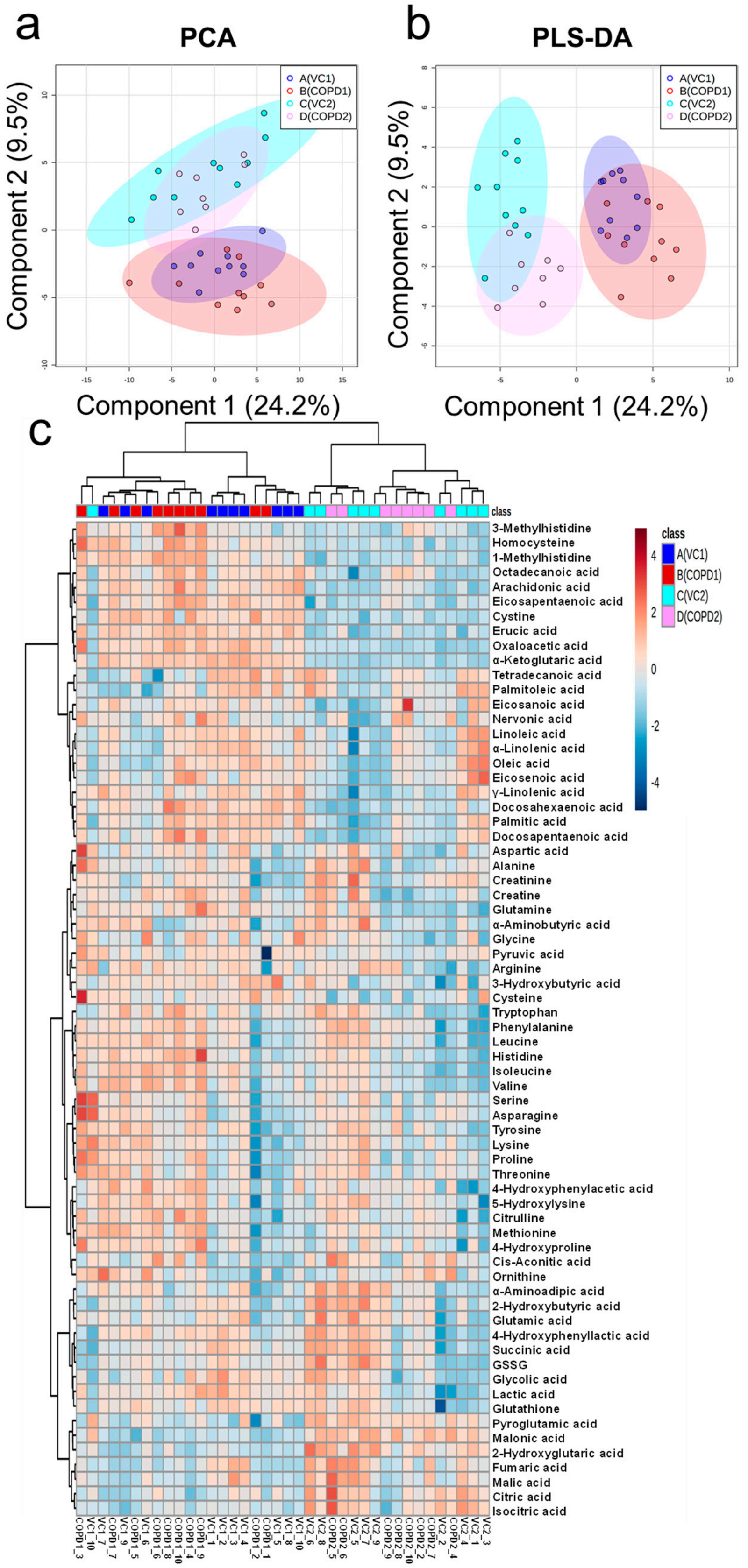

Supplementary Figure S7. PCA scores plot (a), PLS-DA scores plot (b), hierarchical clustering heatmap (c) of plasma in the four groups (COPD1, VC1, COPD2, and VC2).

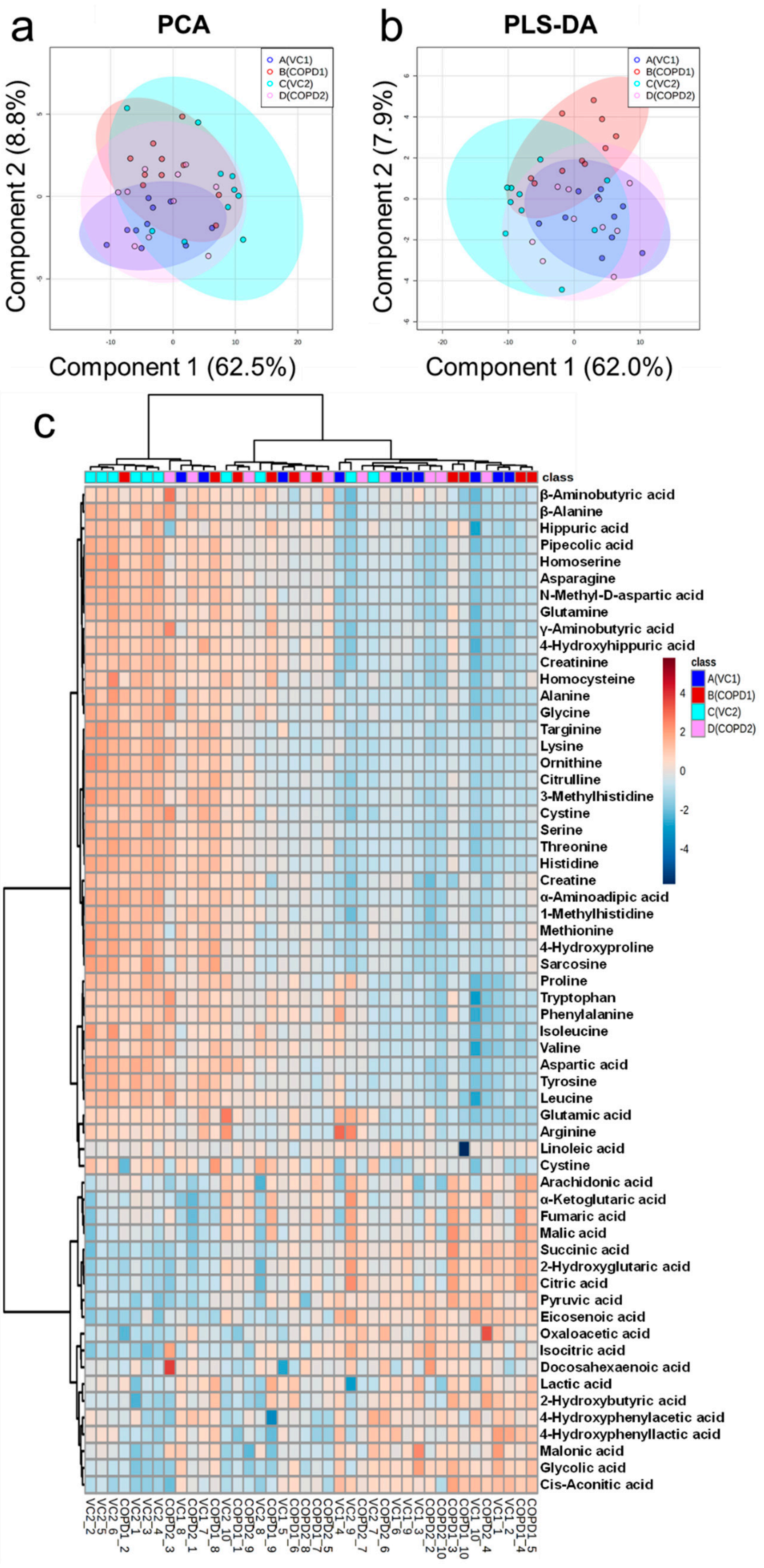

Supplementary Figure S8. PCA scores plot (a), and PLS-DA scores plot (b), hierarchical clustering heatmap (c) of urine in the four groups (COPD1, VC1, COPD2, and VC2).
